# Dynamic Calibration of a Thin-Film Heat-Flux Sensor in Time and Frequency Domains

**DOI:** 10.3390/s22145294

**Published:** 2022-07-15

**Authors:** Zhiling Li, Jianping Yin, Gao Wang, Haijian Liang, Congchun Zhang, Manguo Huang, Yundong Liu, Jie Zhang

**Affiliations:** 1 College of Mechatronics Engineering, North University of China, Taiyuan 030051, China; lizhiling0715@163.com; 2 School of Information and Communication Engineering, North University of China, Taiyuan 030051, China; wanggao@nuc.edu.cn (G.W.); 13223533663@163.com (Y.L.); qwe15513256251@126.com (J.Z.); 3 State Key Laboratory of Dynamic Measurement Technology, North University of China, Taiyuan 030051, China; lianghj@tit.edu.cn; 4 National Key Laboratory of Science and Technology on Micro/Nano Fabrication, Shanghai Jiao Tong University, Shanghai 200240, China; 15835132712@163.com; 5 Aviation Industry Beijing Great Wall Aviation Measurement and Control Technology Research Institute, Beijing 100176, China; 18734118398@163.com

**Keywords:** thin-film heat-flux sensor, dynamic theoretical model, steady-state response, QR decomposition method, transient response, frequency response function, comprehensive evaluation

## Abstract

This paper mainly studies the model design of a thin-film heat-flux sensor (TFHFS), and focuses on the comparison of three dynamic calibration methods. The primary motivation for studying this came from the urgent need for heat-flux dynamic measurements in extreme environments, and the one-sidedness of the dynamic performance evaluation of the corresponding TFHFS. The dynamic theoretical model of the TFHFS was originally established on the principle of a temperature gradient on the basis of a thermal radiation boundary. Then, a novel TFHFS sensor was developed, which can be used at temperatures above 880 °C and has a high sensitivity of 2.0 × 10^−5^ mV/(W/m^2^). It can function stably for long durations under a heat-flux density of 3 MW/m^2^. The steady-state, transient, and frequency calibration of a TFHFS were compared to comprehensively analyze the dynamic characteristics of the TFHFS. The steady-state response time measured by the step excitation method was found to be 0.978 s. The QR decomposition method was applied to the steady-state response experimental model construction, and the fitting degree of a second-order transfer function model obtained was 98.61%. Secondly, the transient response time of the TFHFS was 0.31 ms based on the pulse-excitation method. The transient relationship between the surface temperature and the heat flux, and the pulse-width dependence of the TFHFS transient response time were established. Surprisingly, the response frequency of the TFHFS, about 3000 Hz, was efficiently tested using the frequency response function (FRF), which benefitted from the harmonic characteristics of a periodic square-wave excitation signal. Finally, a comprehensive evaluation of the dynamic performance of the TFHFS was realized.

## 1. Introduction

As a result of the difficulty and importance of heat-flux dynamic testing in the aviation industry, the research purpose of this study is to design a heat-flux sensor that can be applied to the dynamic measurement of heat flux in an extreme environment, and to then make a rational evaluation of its dynamic characteristics. Considering the complexity and the diversity of the currently existing heat-flux dynamic calibration methods and dynamic indicators, it is necessary to compare and analyze the mechanisms and conditions that may be applicable to different calibration methods, in order to comprehensively evaluate the dynamic characteristics of TFHFSs. In the process, construction of both the simulation model and the theoretical model are crucial since both form important initial components for the design of TFHFSs, and are also important later for the evaluation and optimization of such sensors. 

Currently, the primary sensors used in heat-flux dynamic measurements are the thin-film Gardon meter and Atomic Layer Thermopile heat-flux sensors (ALTP). In order to satisfy the assumption of one-dimensional heat conduction, the sensitive element of the Gardon meter, composed of constantan foil, has a radius of more than 5 mm [1]. The Gardon meter is large, and interferes with the surface temperature field; its high-temperature resistance and linearity are poor. Beyond temperatures of 250 °C, the sensitivity of the Gardon heat-flux sensor would decrease, and its non-linearity would increase [2]. As for its dynamic performance, the main concern of this study, the Gardon meter typically has a response time on the order of hundreds of ms [3]. On the other hand, the ALTP heat-flux sensor has high spatial resolution on the order of 1 mm^2^, and excellent dynamic response characteristics, with their fastest responses being up to 1 MHz [4]. Although the sensitivity of this sensor can reach 48 μV/W/mm^2^ [5], it has poor resistance to high temperature, and is therefore prone to damage, making it unsuitable for heat-flux dynamic measurements under harsh conditions. Therefore, these two kinds of heat-flux sensors cannot take into account the working temperature, measurement range, and dynamic performance simultaneously. In contrast, a TFHFS uses microelectromechanical systems (MEMS) technology to optimize the geometric structure and the thermal characteristics of the material. Its high-temperature stability, range of measurement, and dynamic characteristics are superior to those of both the Gardon meter and ALTP heat-flux sensors. This kind of thin-film heat-flux sensor has been reported to tolerate maximum temperatures of up to 1000 °C [6]. Therefore, it has been widely used in heat-flux dynamic measurements of aero-engine hot components. 

In the design and optimization stage of a thin-film heat-flux sensor, its dynamic performance is mainly improved through the application of a reasonable finite element model and an ideal, dynamic theoretical model. When constructing three-dimensional models of TFHFSs, the temperature difference of the thermal insulator layer is used to approximately replace the heat flux, according to Fourier’s law of thermal conductivity [7]. However, the characteristics of this temperature difference, which is proportional to the heat flux, are only valid when the change in the heat flux is lower than a specific frequency; otherwise, greater changes will cause unnecessary errors between simulation and experimental results. In addition, a theoretical model of a heat-flux sensor mostly refers to a thermocouple, which is a typical first-order thermal inertia system. Yet, from a working-principle analysis, a TFHFS, based on a temperature gradient, is essentially different from a thermocouple. Furthermore, the thermoelectric potential measured by a thermopile is inevitably affected by the heat capacity of the substrate, which will reduce the step-response rate of the TFHFS. In contrast, a thermocouple is a single structure, and its heat-transfer mechanism is relatively simple [8]. Considering all of this, for instance, Knauss et al. developed a first-order dynamic model for the ALTP. This model, however, is applicable only to zero-dimensional problems under a thermal convection boundary, but not to a thermal radiation boundary [9]. This means that a new, dynamic theoretical model needs to be developed for TFHFSs only.

In the time domain, dynamic calibration includes steady-state and transient calibrations [10]. The step heat-flux excitation method is usually adopted for the time-domain steady-state calibration of a heat-flux sensor. For example, Cho used a carbon dioxide laser as the heat flux source to complete the steady-state test, but the heat-flux density was low, amounting only to 0.324 MW/m^2^. Besides, the sensor reached a low, steady-state temperature of 650 °C [11]. Guo et al. established a high-order dynamic model of the thin-skin calorimeter [12]. Yang established a transfer-function model of a thin-film thermocouple by the system identification method [13]. On the other hand, the impulse heat-flux excitation method is usually used to obtain the transient-response characteristics of heat-flux sensors in the time domain. For example, Mityakovet et al. performed transient testing of a heat-flux sensor using a low-power NB-YAG laser (50~120 mJ) with a laser pulse width of nanosecond level [14], but a comparison test under different pulse parameters was not performed. Yu et al. [15] used a KRF-excimer laser to test the pulse response of La_0.5_Sr_0.5_COO_3_ thermoelectric film, and found that its rise time was 7 ns. Using this method, a quantitative relationship between the pulse duration and the rise time was obtained. 

In the frequency domain, sinusoidal excitation and square-wave excitation are often used in dynamic calibration studies. In the early frequency response tests, the frequency of the excitation signal was manually adjusted point-by-point, while later instruments mostly adopted the frequency-sweep method. During these tests, the amplitude of the excitation signal was kept unchanged, and the frequency was gradually increased until the measured amplitude–frequency response of the sensors was less than 0.707, or the logarithmic amplitude–frequency was less than −3 dB [16]. Levikari et al. studied the frequency response test of a self-made MEMS heat-flux sensor, and in the experiment, a chopper was used to generate a periodic excitation heat flux of 0~50 Hz. The commercial HFS model of green TEG gSKIN XP was selected as the standard sensor, but due to the limited frequency of the chopper and standard sensor, the test frequency only reached 3.5 Hz [17]. For this reason, the fast Fourier transform (FFT) was used to obtain the response frequency of the heat-flux sensor [18]. The advantage of this method is that only 2~4 frequency points need to be tested, which simplifies the practical steps and improves the calibration efficiency. This periodic thermal problem was also found in industrial areas like blast furnace regenerators and aero-engine cylinders. It is the practical value of heat-flux frequency-domain dynamic testing [19]. 

Based on the above review, this paper adopts thermoelectric multi-physical field coupling in order to build a more effective finite element simulation model, and analyzes the factors that influence the dynamic performance of TFHFSs. MEMS technology is used to fabricate the sensor, which contains 72 R-type thermocouples, effectively improving the sensitivity and the range of the heat-flux sensor. According to the heat-flux testing mechanism for TFHFSs, the dynamic theoretical model of these sensors under the thermal radiation boundary is established.

The calibration methods and the applicable conditions of the steady-state, transient, and frequency calibrations are compared and analyzed through three different modes of heat-flux excitation signals. The steady-state calibration of TFHFSs was completed under ultra-high heat flux, and the second-order transfer-function model with higher accuracy was established by using the QR decomposition method. The factors that influence the transient response of the TFHFS were studied under different pulse excitations. In the frequency calibration, the harmonic characteristics of a periodic square-wave signal were used to improve the calibration efficiency. In other words, the dynamic performance of TFHFSs at high temperatures and large heat fluxes was evaluated more comprehensively. Additionally, some novel principles found in the calibration experiment provided further ideas for the design and dynamic calibration of TFHFSs in the future.

## 2. Design and Preparation of TFHFSs

### 2.1. Structural Design of the TFHFS

**Figure 1:**
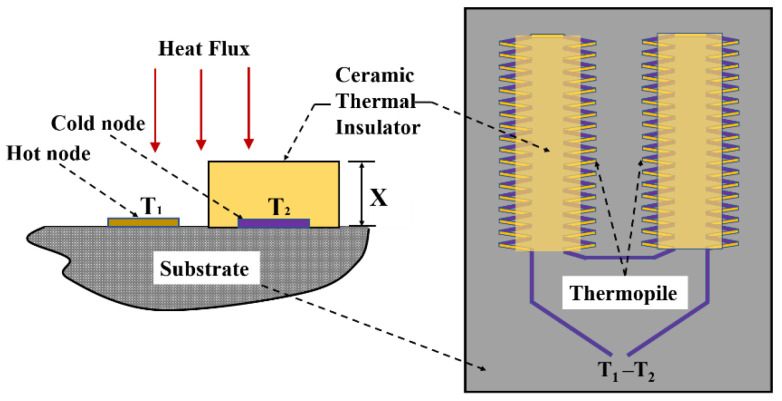
Structural design of the TFHFS used in the present study.

The TFHFS consists of a substrate, a thermopile, and a thermal insulator layer. The thermopile contains 72 thermocouples that convert temperature difference ($T_{1}-T_{2}$) signals into electrical signals based on the Seebeck effect [20]. The structural design is shown in Figure 1. This design converts longitudinal heat flux into a transverse temperature gradient. In heat-flux tests, heat transfer between hot and cold nodes should be avoided as much as possible because it is not directly caused by the heat flux in the normal direction, and will consequently introduce measurement errors.

According to Fourier’s law of heat conduction, the measured heat flux, Q, is proportional to $T_{1}-T_{2}$, as shown Equation (1) [21]. In this relationship, λ and x represent the thermal conductivity of the thermal insulator layer and the thickness of the thermal insulator layer, respectively.

(1) $$Q=\frac{\lambda }{x}\cdot \left(T_{1}-T_{2}\right)$$

### 2.2. Simulation Analysis of Dynamic Characteristics of the TFHFS

**Figure 2:**
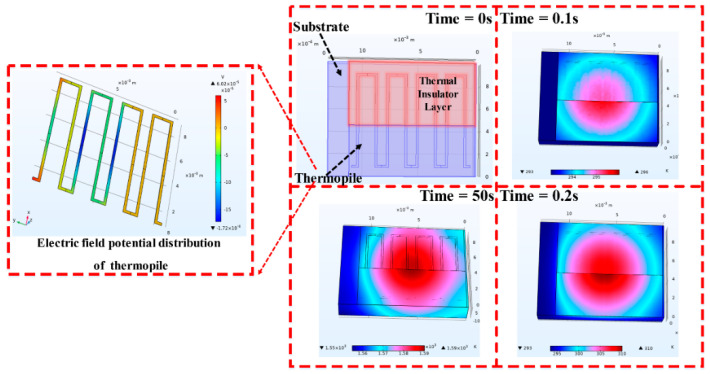
The temperature and the electric–field potential distribution at 3 MW/m^2^ heat flux.

In order to study the factors influencing the dynamic characteristics of the TFHFS, finite element simulation software was used to build a simplified model in 3-D (i.e., in three dimensions). The initial ambient temperature was 20 °C, and the laser provided a thermal load of 3 MW/m^2^. The laser spot radius was 8mm, which can completely cover the sensor, and the laser power was Gaussian, distributed on a two-dimensional plane to fit the actual calibration conditions. At the same time, the natural convection heat dissipation of the air as well as the radiation heat dissipation of the film surface to the environment were taken into account. The convective heat transfer coefficient, h, and the surface emmissivity were 10W/(m^2^·K) and 0.25, respectively. The three-dimensional temperature dynamic process of the TFHFS, and the potential distribution of the thermopile under the above conditions are shown in Figure 2. This simulation is the result of coupling solid heat transfer with the thermoelectric effect. Therefore, the dynamic error caused by the approximate substitution of the temperature difference for heat flux, which occurred in previous studies, was avoided.

**Figure 3:**
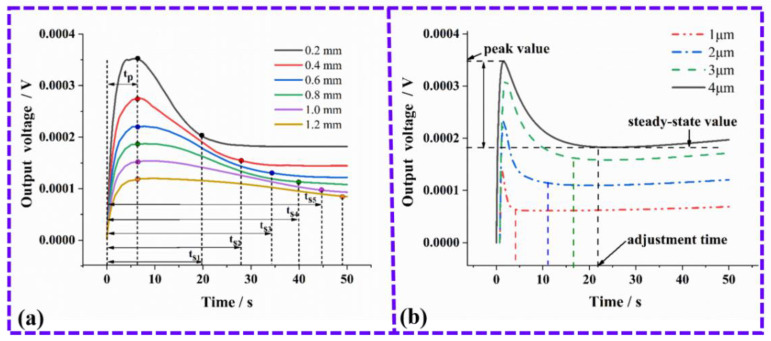
(**a**) Step-response simulation of the TFHFS under different thicknesses of the substrate; (**b**) Step-response simulation of the TFHFS under different thicknesses of the thermal insulator layer.

Based on the previous results of Zhang et al., it is known that the step-response of a TFHFS is independent of the excitation heat flux. Therefore, this research mainly studied the influence of the layer thickness of the thermal insulator, and the thickness of the substrate on the dynamic characteristics of the sample. The step-response simulation of the TFHFS under different substrate thicknesses is shown in Figure 3a. With increasing thickness of the substrate, the adjustment time ($t_{s}$) increases, meaning that the dynamic characteristic of the sensor gets worse. Figure 3b shows the step-response simulation of the TFHFS under different thermal insulator layer thicknesses. It can be seen that under the same heat-flux excitation (3 MW/m^2^), the greater the thickness of the thermal resistance layer, the higher the static sensitivity of the sensor, but the worse the dynamic characteristic. 

By comparing Figure 3a,b, it can also be seen that the thickness of the substrate mainly affects the overshoot (σ). The reason for this is because the back of the substrate is similar to the thermal insulation boundary, which hinders the longitudinal conduction of the heat flux, resulting in a more significant heat-transfer effect from the hot node to the cold node. Therefore, on these bases, it can be inferred that the output of the TFHFS falls back after reaching its peak. In fact, the substrate only acts as a carrier, and simulates the object being tested. In the heat-flux test of turbine blades, the thermopile and the thermal insulator layers need to be deposited directly on the turbine blades in large volumes, in order to make conditions closer to the heat conduction of a semi-infinite body. Its characteristic is that the dynamic heat conduction is only along the vertical direction, and the proportion of the transverse heat conduction can be greatly reduced.

### 2.3. Preparation of the TFHFS

**Figure 4:**
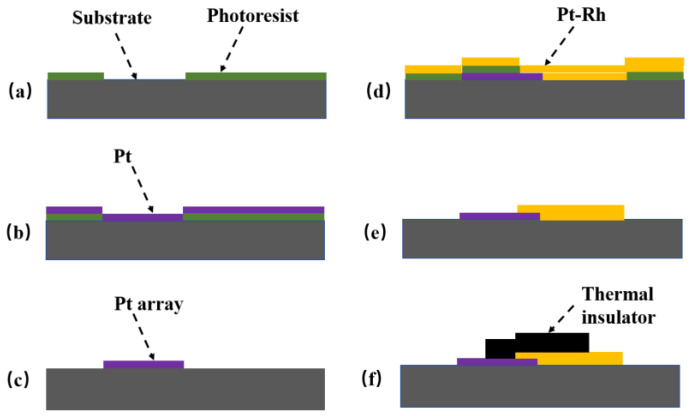
Preparation process of the TFHFS (**a**–**f**). (**a**) A layer of photoresist was sputtered onto the substrate; (**b**) the 0.3-micrometer Pt electrode was sputtered; (**c**) the photoresist was removed; (**d**) the 0.3-micrometer Pt-Rh13 electrode was sputtered; (**e**) the photoresist was removed again; (**f**) the thermal insulator layer was deposited with a thickness of 2.5 μm.

**Figure 5:**
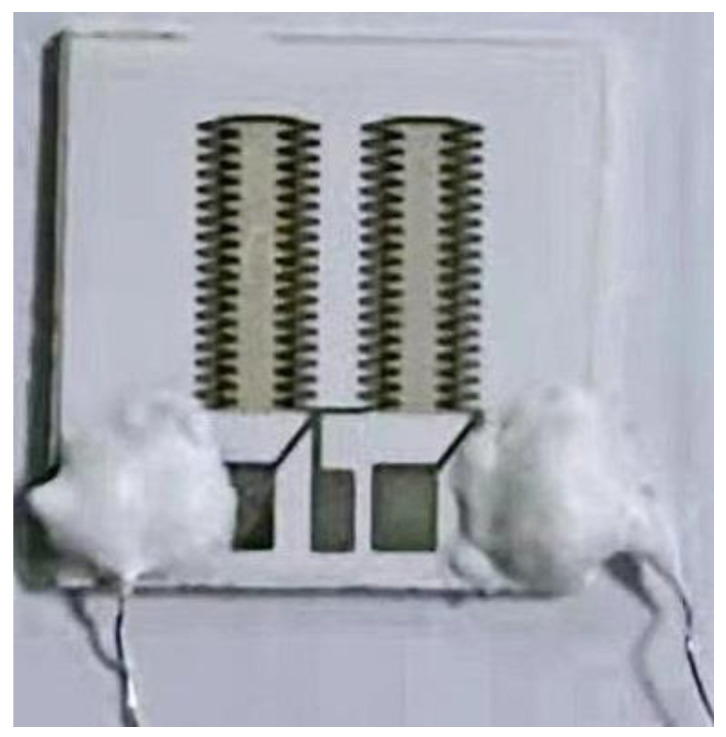
Physical picture of the TFHFS.

**Figure 6:**
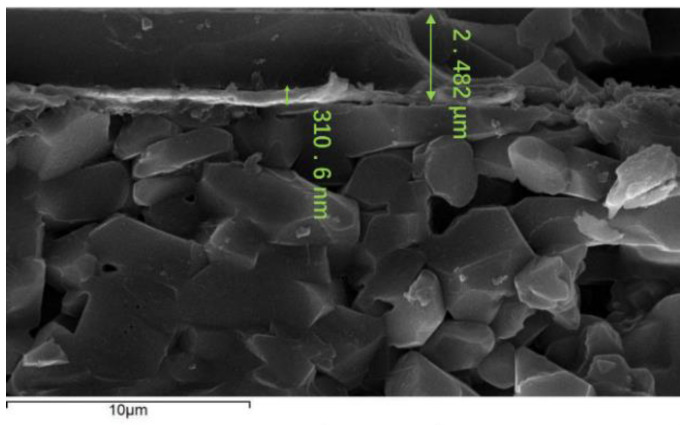
Layer thicknesses of the TFHFS.

The TFHFS was fabricated using MEMS technology. Silicon dioxide was chosen as the thermal insulator, and the substrate material was alumina. The thermopile materials used were platinum and platinum-13% rhodium. The film was imprinted via microlithography and stripping, as shown in Figure 4. The physical picture of the sample and the characterization results of the film thickness are shown in Figure 5 and Figure 6, respectively. A preliminary static calibration experiment showed that its sensitivity was 2.0 × 10^−5^ mV/(W/m^2^).

Compared with previous sensor designs, the sensor’s thermopile in this study adopted a rectangular structure. More thermocouples were sputtered onto a smaller substrate, which improved the sensitivity coefficient of the sensor. As a result of film materials and dimensional parameters, excellent high-temperature resistance and dynamic characteristics are guaranteed. Parameters of the same type of the thin-film heat-flux sensor are shown in Table 1. Compared with similar sensors, the TFHFS has a higher sensitivity, and the range of heat-flux measurements of the sensor reached 3 MW/m^2^, according to the steady-state calibration experiment. The sensor could also withstand temperatures of more than 880 °C. Therefore, the performance indexes of the sensor are relatively balanced, and can be applied to dynamic heat-flow testing in complex thermal environments.

## 3. Dynamic Calibration Experiment in Time Domain

### 3.1. Steady-State Calibration Experiment

#### 3.1.1. Theoretical Modeling under Thermal Radiation Boundary

Previously, traditional dynamic theoretical models referred to the thermocouple, and also tended to ignore the temperature gradient principle of a TFHFS. In addition, these previous models were based on a lumped parameter method. This theory only applied to the thermal convection boundary, but this present study proposes the dynamic theoretical model of a TFHFS under a thermal radiation boundary. According to Equation (1), the dynamic response of a TFHFS can be approximately replaced by the temperature difference of the thermal insulator layer. When heated by a constant heat flux of laser radiation, the governing equations and the definite solution condition of the temperature field of the thermal insulator layer are hereby shown in Equation (2):

(2) $$\frac{\partial T}{\partial t}=a\frac{\partial^{2}T}{\partial x^{2}}\;,\;0<x<\infty \;t=0,\;T\left(x,t\right)=T_{0}\;x=0,-\lambda \frac{\partial T}{\partial x}=q_{0}$$

With the second type of the thermal boundary, the solution of temperature field of thermal insulator layer is as follows:

(3) $$T\left(x,t\right)=T_{0}+\frac{2q_{0}\sqrt{\frac{at}{\pi }}}{\lambda }\exp\left(-\frac{x^{2}}{4at}\right)-\frac{q_{0}x}{\lambda }erfc\left(\frac{x}{2\sqrt{at}}\right)$$

Therefore, the hot-node temperature $T_{1}$ is determined as follows:

(4) $$T_{1}=T_{0}+\frac{2q_{0}\sqrt{\frac{at}{\pi }}}{\lambda }$$

The cold-node temperature $T_{2}$ is defined as follows:

(5) $$T_{2}=T_{0}+\frac{2q_{0}\sqrt{\frac{at}{\pi }}}{\lambda }\exp\left(-\frac{x^{2}}{4at}\right)-\frac{q_{0}x}{\lambda }erfc\left(\frac{x}{2\sqrt{at}}\right)$$

Then, the analytical solution of the TFHFS dynamic response is determined as follows:

(6) $$\Delta T=T_{1}-T_{2}=\frac{2q_{0}\sqrt{\frac{at}{\pi }}}{\lambda }\left[1-\exp\left(-\frac{x^{2}}{4at}\right)\right]+\frac{q_{0}x}{\lambda }erfc\left(\frac{x}{2\sqrt{at}}\right)$$

where $q_{0}$, x, a, and erfc are the heat-flux density of laser radiation, the thickness of the thermal insulator layer, the thermal diffusion coefficient of the thermal insulator layer, and the error remainder function, respectively. When the laser parameters, the structural dimensions, and the thermal and physical properties of the TFHFS are known, the dynamic response curve of the sensor can be obtained.

**Figure 7:**
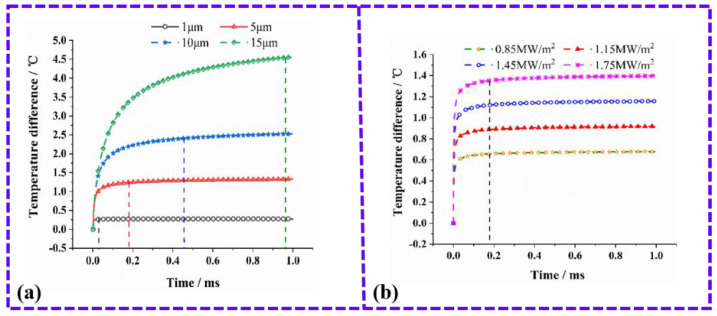
(**a**) Verification results of the theoretical model with different thicknesses of the thermal insulator layer; (**b**) Verification results of the theoretical model with different heat–flux densities and the 2.5–micrometer thermal-insulator layer.

When the laser power and the spot radius were 600 W and 15 mm, respectively, the heat-flux density was 0.85 MW/m^2^. The conditions and parameters of this theoretical model are shown in Table 2, and the model is consistent with the previous finite element simulation results. When a TFHFS is deposited directly on the surface of an object without any substrate, the dynamic characteristics of the sensor are only determined by the thickness of the layer of the thermal insulator. The theoretical model described in Equation (6) was verified by numerical simulation. According to Equation (1), the dynamic response of the TFHFS can be approximately replaced by temperature difference $T_{1}-T_{2}$. The verification results are shown in Figure 7. This theoretical model describes the one-dimensional heat transfer of a semi-infinite body in general, and needs not consider the transverse heat conduction caused by the substrate, so that there is no overshoot phenomenon. According to the actual parameters of the TFHFS, $T_{r}$ of the sensor is 98 μs, where $T_{r}$ is the steady-state response time, which represents the time to reach 95% of the steady-state value of the step response. 

#### 3.1.2. Steady-State Calibration Experiment

**Figure 8:**
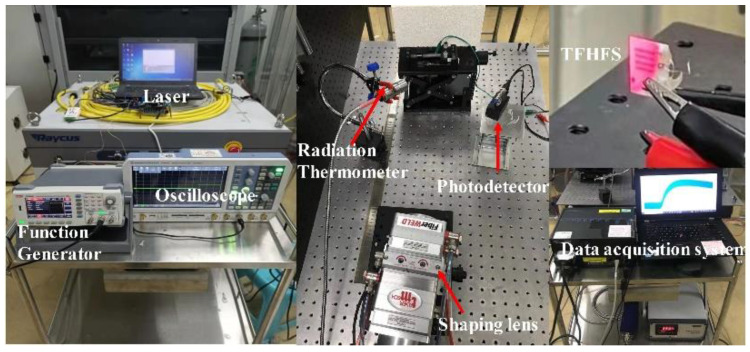
The laser radiation heat-flux dynamic test system used in the present study.

The test device includes a semiconductor laser, a function generator, a beam shaping lens set, a high-speed infrared radiation thermometer, a THORLABS DET10A/M silicon photodetector (with a rise time and wavelength of 200–1100 nm, respectively), and a data acquisition system, as shown in Figure 8. In this experiment, it was assumed that the transverse heat conduction from the hot node to the cold node of the TFHFS can be neglected compared with the longitudinal excitation of large heat flux. The high-power semiconductor laser was used as a dynamic heat-flux source. The laser had a maximum output power of 6000 W, and a wavelength of 915 nm located in the infrared region. The function generator can modulate the output mode of the laser, and the laser beam can illuminate the sample surface through the quasi-lens group. Finally, the surface temperature of the TFHFS was collected using a high-speed infrared radiation thermometer in real time.

**Figure 9:**
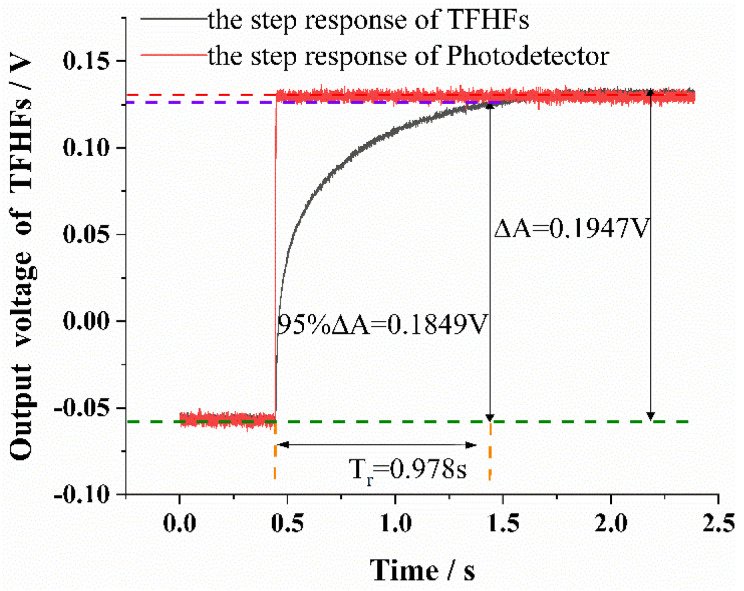
Experimental curve of the step response of the TFHFS.

Setting the laser power, P, and the spot radius to 600 W and 8 mm, respectively, the heat-flux density, $q_{1}$, was found to be 3 MW/m^2^. The step laser was modulated by a function generator. When the output of the TFHFS reaches a steady-state value, the step response amplitude ΔA was recorded, followed by calculation of the steady response time, $T_{r}$. The experimental results are shown in Figure 9, and it can be seen from the experimental curve that the rising edge of the laser signal measured by the photodetector is close to the ideal step signal. The amplitude, ΔA, and the steady-state response time, ${T^{\prime }}_{r}$, were 0.1947 V and 0.978 s, respectively. Compared with the simulation results in Figure 3, the step response of the TFHFS did not overshoot.

It was found that ${T^{\prime }}_{r}$ obtained through the step-response experiment was much larger than the theoretical value predicted from the theoretical model in Section 3.1.1. The reason for this is because the theoretical model was based on a semi-infinite body assumption, but the TFHFS was deposited onto the substrate material, which was not suited for ideal one-dimensional heat conduction. At high temperatures, especially, the thermal conductivity, λ, of the film will become worse. Moreover, it must also be taken into account that the sensor is a multilayer thin-film structure, and that the interlayer thermal resistance has a great influence. All of these factors determined that the experimental value, ${T^{\prime }}_{r}$, was greater than the theoretical value,$\;T_{r}$. In addition, the thermal properties of micro/nano films are quite different from those of the materials whose sizes were more conventional. 

#### 3.1.3. Experimental Modeling under a Thermal Radiation Boundary

The system identification method was carried out to get a more accurate transfer function model, according to the experimental data the of TFHFS step response shown in Figure 9. The data collected by the photodetector served as the system input,  x(n), and the thermoelectric potential of TFHFS served as the system output,  y(n), with a sampling interval of 0.000625 s. Resampling, in the proportion 1:30, of the original data was carried out to eliminate noise interference, and hence the sampling interval was 0.01875 s. With a bandwidth of about 5 MHz, the photodetector was assumed to have a flat frequency response in the target band, and the phase lag was negligible. In this case, it represented the heat-flux input signal of the TFHFS [22].

A TFHFS can be regarded as a linear time-invariant system with single inputs and outputs, which can thus be described as follows by a differential equation of type Equation (7):

(7) $$A\left(d^{-1}\right)y\left(k\right)=B\left(d^{-1}\right)u\left(k\right)+\varepsilon \left(k\right)$$

where u(k) and y(k) are the input and output of the system, respectively, ε(k) is the fitting error,$\;d^{-1}$ is the post-shift operator, and n is the model order. The subsequent relations are defined as follows in Equations (8)–(10):

(8) $$d^{-1}y\left(k\right)=y\left(k-1\right)$$

(9) $$A\left(d^{-1}\right)=1+a_{1}d^{-1}+a_{2}d^{-2}+\ldots +a_{n}d^{-n}$$

(10) $$B\left(d^{-1}\right)=b_{0}+b_{1}d^{-1}+b_{2}d^{-2}+\ldots +b_{n}d^{-n}$$

In the model-system identification of a TFHFS, the model order, n, was determined according to the step-response experimental data. First, the information matrix, D, was established according to the measured data, as shown in Figure 9.

$$D={\left[\begin{array}{cccccc} u\left(1\right) & -y\left(1\right) & u\left(2\right) & -y\left(2\right)\cdots & \;u\left(v+1\right) & -y\left(v+1\right)\\ u\left(2\right) & -y\left(2\right) & u\left(3\right) & -y\left(3\right)\cdots & \;u\left(v+2\right) & -y\left(v+2\right)\\ \vdots & \vdots & \vdots & \vdots & \vdots & \vdots \\ u\left(N\right) & -y\left(N\right) & u\left(N+1\right) & -y\left(N+1\right)\cdots & u\left(v+N\right) & -y\left(v+N\right) \end{array}\right]}_{N\times M}$$

The orthogonal transformation of D can lead to the upper triangular matrix, R, which is shown in Table 3, and the red numbers represent the residuals of order 1 to 6.

According to the QR decomposition method, the sum of the squares of the even diagonal elements of matrix R is equal to the sum of squares of residuals J(n) of different orders, for which the calculation results are as follows:

$$\begin{array}{ccc} J\left(1\right)=0.00003196 & J\left(2\right)=0.00000778 & J\left(3\right)=0.00000749\\ J\left(4\right)=0.00000713 & J\left(5\right)=0.00000684 & J\left(6\right)=0.00000640 \end{array}$$

**Figure 10:**
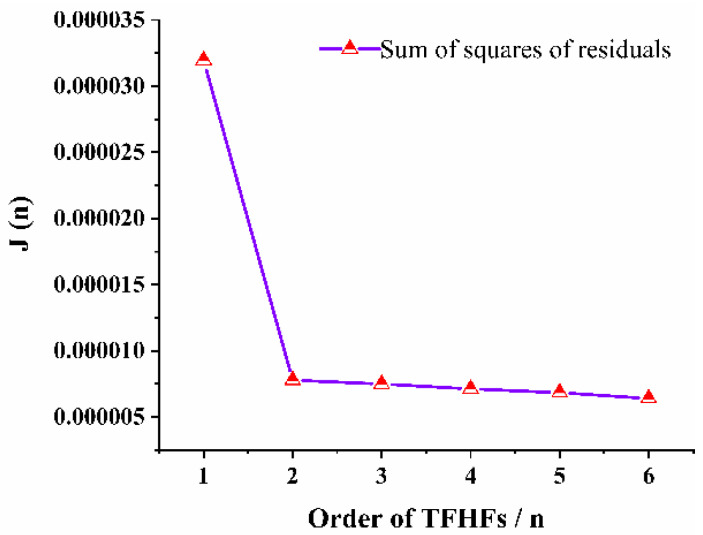
Comparison of the residuals curves of the TFHFS model.

**Figure 11:**
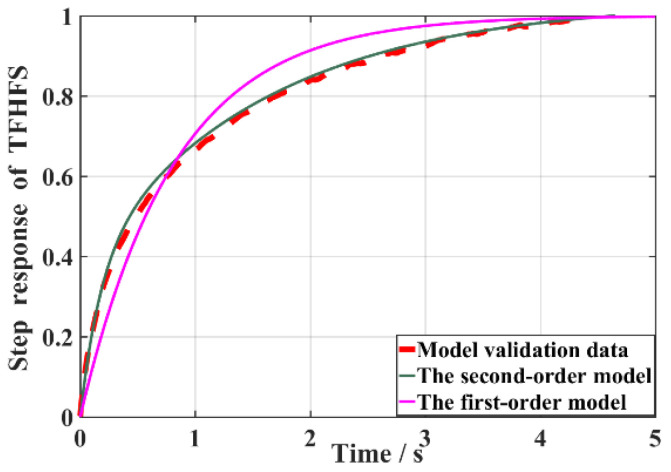
Verification of the fitting degree.

According to the variation trend of J(n) with order n, J(2) decreased greatly compared with J(1), while J(3), J(4), J(5), and J(6) changed relatively little, as shown in Figure 10. Using the model-order estimation criterion based on the sum of squares of residuals, it can be concluded that the model order of the TFHFS is 2. Since the QR decomposition method obtained the order and the model parameters of the system at the same time, it was easy to obtain the transfer function model of the TFHFS, as shown in Equation (11). If the TFHFS is regarded as a first-order system, then the transfer function model obtained by using the same method is $H^{\prime }\left(s\right)$, as shown in Equation (12). Compared with the model validation data, the fitting degree of the second-order model is 98.61%, while that of the first-order model is only 82.54%, as shown in Figure 11. Therefore, the second-order transfer function model established for the TFHFS is more reasonable than the traditional first-order model.

(11) $$H\left(s\right)=\frac{2.554s+3.68}{s^{2}+6.304s+3.552}$$

(12) $$H^{\prime }\left(s\right)=\frac{1.224}{s+1.224}$$

Referring to classical control theory for a second-order system, its steady-state response time, $T_{r}$, is inversely proportional to the product of the natural frequency, $\omega_{n}$, and the damping coefficient, ζ, as shown in the following Equation (13):

(13) $$T_{r}=3/\omega_{n}\zeta$$

According to the results of the transfer function, H(s), as shown before, it may be deduced that $\omega_{n}\zeta =3.152$. The steady-state response time of the TFHFS is 0.978 s, as shown in Figure 9, and calculations results are shown in Equation (14). The calibration result of the TFHFS basically conforms to empirical Equation (13), with an error of only 2.6%, which shows that it is reasonable to regard the calibrated sensor as a second-order system. In addition, this conclusion also serves to obtain the response time directly from the transfer function model of the heat-flux sensor, which improves the calibration efficiency, and may thus be of benefit in future applications.

(14) $$T_{r}=0.978\;s\;3/\omega_{n}\zeta =0.952\;s$$

### 3.2. Transient Calibration Experiment

#### 3.2.1. Impulse Response Experiment of Surface Temperature and Heat Flux

In order to optimize the thermal protection design of aero-engine hot-end components, it is necessary to explore the transient relationship between the surface heat flux and the surface temperature under a large heat-flux impact state. With this in mind, the experimental pulse width of the modulated laser, the laser power, and the heat flux density were 5 ms, 6000 W, and 30 MW/m^2^, respectively. With these parameters, the surface temperature of the TFHFS was then detected by a high-speed infrared radiation thermometer, and a photodetector detected the laser signal.

**Figure 12:**
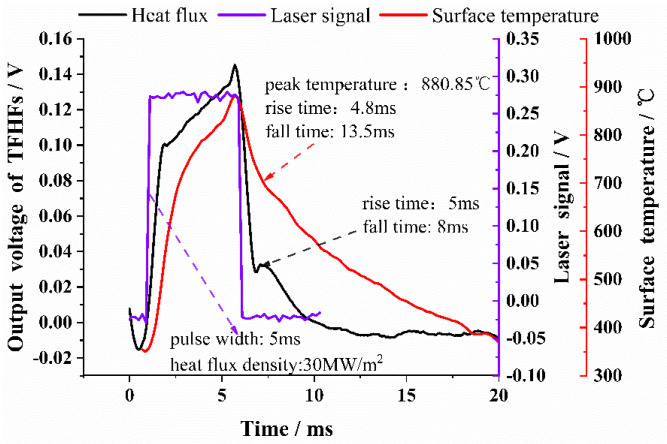
The dynamic response of the surface temperature and the heat flux.

The test results are shown in Figure 12. The peak temperature of the sample surface reached 880.85 °C. The rise time of the heat-flux signal was roughly the same as that of the surface temperature; however, the hysteresis of the heat flux was also less than that of the surface temperature, and the rising edge of the heat flux was steeper than that of the latter in the early response period. Moreover, the decay time of the surface temperature (13.5 ms) was significantly slower than that of the heat flux (8 ms). This also means that the rising edge was faster for the heat-flux signal than the falling edge. It became necessary to analyze the mechanism behind the phenomenon that caused the surface heat flux to be ahead of the surface temperature. Toward this, the theory of periodic transient heat conduction in heat transfer can be used for reference: for instance, when the surface temperature of a semi-infinite body changes periodically, its surface heat flux also changes according to the simple harmonic law, and the surface heat-flux wave is one phase ahead of the temperature wave [23]. This explains why the transient response of the TFHFS is ahead of the surface temperature, as shown in Figure 12.

Previous studies ignored the reference function of the falling edge in the evaluation of the dynamic index of the heat-flux sensor. In fact, it is more meaningful to use the falling edge to describe the dynamic characteristics of a TFHFS because it represents how quickly a TFHFS recovers from the peak heat flux to the initial state. When the sensor was applied to the hot component of an aero-engine, it quickly recovered to a lower initial value. This is clearly an advantage in the field of dynamic heat-flux testing [24].

#### 3.2.2. Impulse Response Experiment of the TFHFS

**Figure 13:**
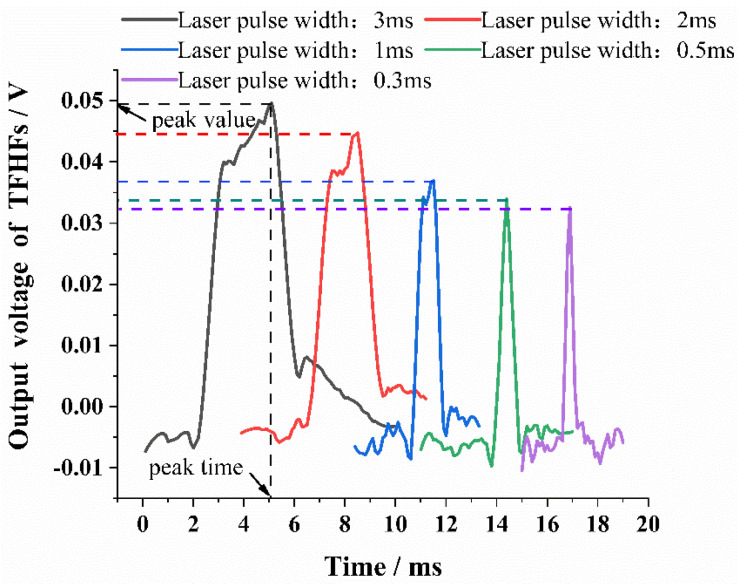
Impulse response of TFHFS at different pulse widths.

In order to test the transient response characteristic of the TFHFS, the laser power was reduced to 3000 W, and the heat-flux density was $q_{3}=15\;\mathrm{MW}/m$^2^. Due to the large number of experiments, the laser power was reduced in order to avoid the sample being damaged. In the experiment, the function generator was successively used to modulate the pulsed laser signal of 3 ms, 2 ms, 1 ms, 0.5 ms, and 0.3 ms to complete the transient calibration of the TFHFS. The test curves are shown in Figure 13, and the results show that under the same laser power, there exists a direct relationship between the pulse width and the amplitude and the transient response time ($T_{p}$). When the pulse width decreased to 0.3 ms, the sample did not respond in time. Experimental results showed that the transient response time of the TFHFS, under the impact of a large heat flux, can reach 0.31 ms under the existing test conditions, as shown in Figure 13. 

The experimental results show that the transient response time of the TFHFS is dependent on the width of the laser pulse. The reason for this is that under the same laser power, P, the narrower the width of laser pulse, $\tau_{p}$, the smaller the pulse energy, Q, as is described in Equation (15). This results in less thermal energy being converted to the thermoelectric output of the TFHFS, and further shows that under the same heat-flux density, the width of the laser pulse affected the amplitude of the TFHFS, which in turn affected the transient response time of the sample. Therefore, when using pulsed heat flux to calibrate the TFHFS, or when comparing transient response characteristics of different sensors, the width and power of the excitation signal should be stated, which is of benefit for error assessment.

(15) $$Q=P\times \tau_{p}$$

From another point of view, the rising stage of the TFHFS impulse response is a forced process. Its attenuation stage, however, is a natural cooling process, which reflects the inherent transient characteristic of the sensor. If the attenuation time of the TFHFS was not affected by the parameters of a pulse, then an evaluation of the transient response of thin-film heat-flux sensors could have been performed. Needless to say, this must be further examined, and may require verification from a larger set of experiments. 

## 4. Dynamic Calibration Experiment in Frequency Domain

In the field of aeronautics and astronautics, it is necessary to know the response frequency of a heat-flux sensor when measuring high-frequency thermal disturbances. This paper used a periodic square-wave signal as an excitation signal; a photoelectric detector was used to record laser signal as system input, and a TFHFS output was used as system output. Finally, the response frequency was calculated using a frequency response function (FRF) as shown in Equation (16):

(16) $$H\left(f\right)=\frac{Y_{out}\left(f\right)}{X_{in}\left(f\right)}$$

where $X_{in}\left(f\right)$ is the fast Fourier transform (FFT) of the heat-flux input signal from the photoelectric detector, and $Y_{out}\left(f\right)$ is the FFT of the heat-flux output signal from the TFHFS. It should be emphasized here that the laser signal collected by the photodetectors did not represent the absolute amplitude of the heat-flux input signal. Therefore, the frequency response function of the TFHFS needed to be normalized to the amplitude at the fundamental experimental frequency, as shown in Equation (17):

(17) $$H_{n}\left(f\right)=H\left(f\right)/H_{0}\left(f\right)$$

Here, $H_{0}\left(f\right)$ is the amplitude at the fundamental frequency, and H(f) is the amplitude at the harmonic frequency derived from the FFT. When $H\left(f\right)/H_{0}\left(f\right)$ decayed to 0.707, which was equivalent to a prescribed attenuation of −3 dB, the corresponding harmonic frequency was the response frequency of the TFHFS.

**Figure 14:**
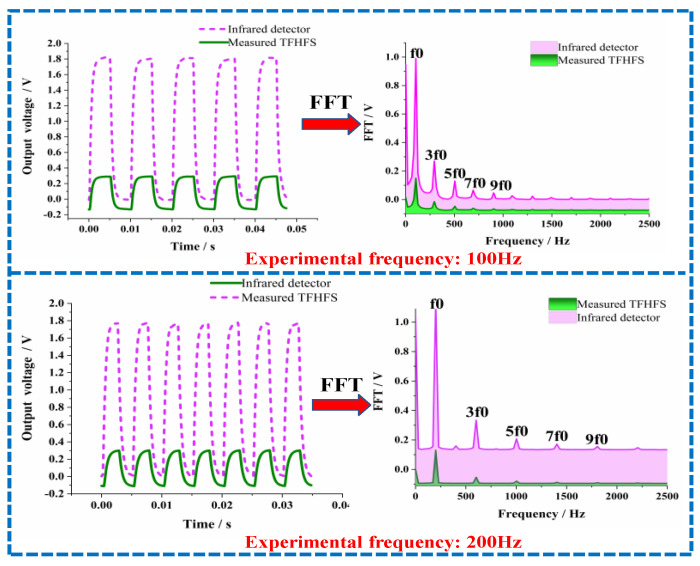
The time series and the spectrum distribution at different square-wave frequencies.

This study used 100-Hertz and 200-Hertz laser signals to complete the dynamic calibration experiment in a frequency domain. The heat-flux density was 3 MW/m^2^, and the sampling rate was 10,000 Hz. The sample time series and spectral distribution under two different square-wave frequencies are shown in Figure 14. The symmetrical periodic square-wave heat-flux signal was used in the experiment, whose pulse width was exactly half of the period. For the ideal symmetric periodic square-wave signal, after Fourier transform, the even harmonics of the signal fell to value zero of the spectrum envelope, so that its spectrum contained only fundamental and odd harmonics components. The experimentally obtained Fourier amplitudes of the even harmonics were not zero but close to it, as shown in Figure 14.

**Figure 15:**
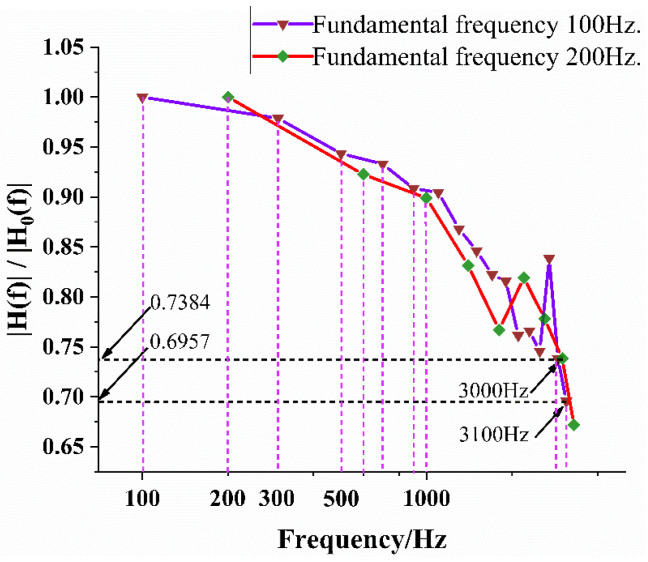
Amplitude–frequency response characteristic curve at different square-wave frequencies.

According to the FRF method, as shown in Equations (16) and (17), the calculation process of the amplitude–frequency response is shown in Table 3, taking the experimental data of 200 Hz as an example. When $H\left(f\right)/H_{0}\left(f\right)$ attenuated to 0.707, the response frequency, f, of the TFHFS was in the range of 3000 Hz to 3400 Hz. In order to further confirm the response frequency, the experimental frequency was adjusted to 100 Hz. Using the same method, the response frequency of the TFHFS could further be determined to be between 2900~3100 Hz. Based on the data in Table 4, the amplitude–frequency response characteristic curves of the two groups of experiments are plotted in Figure 15.

The response frequency, f, of the TFHFS was in the range of 3000 Hz to 3100 Hz, which indicated that the TFHFS ensured that the amplitude frequency error was lower than 0.293 when the thermal disturbance was tested with a frequency lower than 3000 Hz. Therefore, using the harmonic characteristics of a periodic square-wave signal can greatly reduce the number of experiments and improve the calibration efficiency. If periodic sinusoidal signals are used as heat-flux excitation, point-by-point tests from low to high frequencies are required. 

## 5. Conclusions

In this paper, a thin-film heat-flux sensor was fabricated using MEMS technology. The requirements of high-temperature resistance, the dynamic characteristics, and the sensitivity of the TFHFS in an extreme thermal environment were simultaneously considered. Results show that the sensor could withstand the highest and tested temperature of 880 °C, and had a static sensitivity of 2.0 × 10^−5^ mV/(W/m^2^). Moreover, the sensor functioned stably at a heat flux of 3 MW/m^2^ for a relatively long duration. Through the dynamic calibration in the time domain, the steady-state response time, $T_{r}$, and the transient response time, $T_{p}$, of the sensor were 0.978 and 0.31 ms, respectively. The response frequency, f, reached about 3000 Hz by calibrating the frequency domain. By comparing the three different dynamic excitation signals as used here, the finding are as follows:

(1) In the finite element simulation, the thickness of the substrate affects the overshoot of the TFHFS’s dynamic response because the substrate is not an ideal semi-infinite body, which further affects the one-dimensional heat conduction of the heat flux.
(2) The dynamic theoretical model of a TFHFS should take into consideration its temperature gradient principle and thermal radiation boundary condition.
(3) The second-order transfer function model of the TFHFS identified by the QR decomposition method had a higher accuracy than the first-order thermal inertia model in steady-state calibration. In addition, the parameters of the second-order model ($\omega_{n},\zeta$) could be quantitatively transformed with steady-state time, $T_{r}$.
(4) In the transient calibration, the surface heat flux of the TFHFS was always ahead of the surface temperature, regardless of the rising or the falling edge of the impulse response. Moreover, the calibration results depended on the width of the pulse of the excitation signal.
(5) In the frequency domain calibration, the periodic square excitation signal was more efficient than the sinusoidal excitation signal because of its harmonic characteristics.

In summary, this study obtained the dynamic characteristics of a TFHFS under different thermal conditions of aero-engines (aero-engine start-up, large heat-flux impacts, and high-frequency thermal disturbances), which demonstrated significant practical value. In the future, it is yet still necessary to study dynamic models with higher accuracy for such sensors, and to further analyze the internal relationships and differences in the dynamic response characteristics of these sensors under different heat-flux excitations.

## Figures and Tables

**Table 1:** Comparison of indexes of film heat-flux sensors.

Research Institutions	Electrode Materials	Number of Thermocouples	Sensitivity/(mV/W/m^2^)	Operating Temperature/(°C)	Measurement Range/(kW/m^2^)
Beijing University of Technology	Cu/Ni	9	1.01 × 10^−5^	-	0.7
Xiamen University	ITO/In_2_O_3_	40	6.1 × 10^−5^	888	236.4
Huazhong University of Science and Technology	W-5Re/W-26Re	12	3.8 × 10^−6^	1000	1000
Western Michigan University	Pt/PtRh10	40	1.2 × 10^−7^	800	324
This study	Pt/PtRh13	72	2.0 × 10^−5^	880	3000

**Table 2:** Conditions and parameters of the theoretical model.

$q_{0}$	*a*	λ	*x*
0.85 MW/m^2^	0.968 × 10^−6^ m^2^/s	3.06 W/(m K)	2.5 μm

**Table 3:**
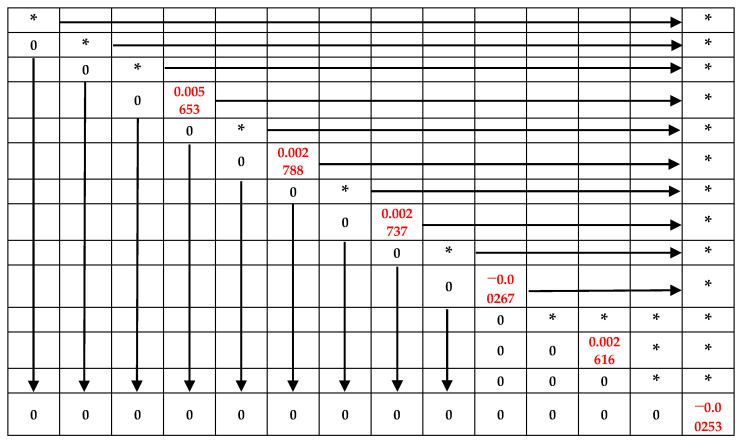
The upper triangular matrix, *R*.

**Table 4:** The FRF analyzed on the basis of an experimental frequency of 200Hz.

f(Hz)	Xin(f)	Xin(f)/Xin0(f)	Yout(f)	Yout(f)/Yout0(f)	H(f)/H0(f)
**200**	1.0066	1	0.2244	1	1
**600**	0.2014	0.2000	0.0414	0.1845	0.9225
**1000**	0.0748	0.0743	0.0150	0.0668	0.8990
**1400**	0.0389	0.0386	0.0072	0.0321	0.8316
**1800**	0.0227	0.0226	0.0039	0.0174	0.7670
**2200**	0.0145	0.0144	0.00267	0.0118	0.8194
**2600**	0.00986	0.00979	0.00171	0.00762	0.7783
**3000**	0.00705	0.00700	0.00116	0.05169	0.7384
**3400**	0.00504	0.00500	0.000756	0.00336	0.6720

## Nomenclature

$T_{r}$	steady-state response time (the time to reach 95% of the steady-state value of the step response)/s
$t_{s}$	adjustment time (the time when the steady-state error of the step response of the second-order system enters the range of ±0.05 or ±0.02)/s
$T_{p}$	transient response time (the time to reach 100% of the peak value)/s
$\tau_{p}$	pulse width (duration of pulse heat-flux excitation signal)/s
$\omega_{n}$	natural frequency of second-order system/Hz
ζ	damping coefficient of the second-order system
$t_{p}$	peak time (the time required for the step response of a second-order system to reach the first peak value)/s
ΔA	amplitude of the step response/V
f	response frequency/Hz
